# Quantitative Analysis of Real-Time Infrared Thermography for the Assessment of Lumbar Sympathetic Blocks: A Preliminary Study

**DOI:** 10.3390/s21113573

**Published:** 2021-05-21

**Authors:** Mar Cañada-Soriano, José Ignacio Priego-Quesada, Maite Bovaira, Carles García-Vitoria, Rosario Salvador Palmer, Rosa Cibrián Ortiz de Anda, David Moratal

**Affiliations:** 1 Applied Thermodynamics Department (DTRA), Universitat Politècnica de València, 46022 Valencia, Spain; macaaso@upv.es; 2 Research Group in Sports Biomechanics (GIBD), Department of Physical Education and Sports, University of Valencia, Avda. Blasco Ibáñez, 15, 46010 Valencia, Spain; 3 Research Group in Medical Physics (GIFIME), Department of Physiology, University of Valencia, Avda. Blasco Ibáñez, 15, 46010 Valencia, Spain; Rosario.Salvador@uv.es (R.S.P.); rosa.m.cibrian@uv.es (R.C.O.d.A.); 4 Anaesthesia Department, Hospital Intermutual de Levante, Sant Antoni de Benaixeve, 46184 Valencia, Spain; bovaira@gmail.com (M.B.); carlesgvitoria@gmail.com (C.G.-V.); 5 Center for Biomaterials and Tissue Engineering, Universitat Politècnica de València, 46022 Valencia, Spain

**Keywords:** complex regional pain syndrome, skin temperature, thermal image, lower limbs, regional anesthesia

## Abstract

Lumbar sympathetic blocks (LSBs) are commonly performed to treat pain ailments in the lower limbs. LSBs involve injecting local anesthetic around the nerves. The injection is guided by fluoroscopy which is sometimes considered to be insufficiently accurate. The main aim was to analyze the plantar foot skin temperature data acquired while performing LSBs in patients with complex regional pain syndrome (CRPS) affecting the lower limbs. Forty-four LSBs for treating lower limb CRPS in 13 patients were assessed. Pain medicine physicians visualized the infrared thermography (IRT) video in real time and classified the performance depending on the observed thermal changes within the first 4 min. Thirty-two percent of the cases did not register temperature variations after lidocaine was injected, requiring the needle to be relocated. Differences between moments are indicated using the 95% confidence intervals of the differences (CI 95%), the Cohen effect size (ES) and the significance (*p* value). In successful cases, after injecting lidocaine, increases at minute 7 for the mean (CI 95% (1.4, 2.1 °C), *p* < 0.001 and ES = 0.5), at minute 5 for maximum temperature (CI 95% (2.3, 3.3 °C), *p* < 0.001 and ES = 0.6) and at minute 6 for SD (CI 95% (0.2, 0.3 °C), *p* < 0.001 and ES = 0.5) were observed. The results of our preliminary study showed that the measurement of skin temperature in real time by infrared thermography is valuable for assessing the success of lumbar sympathetic blocks.

## 1. Introduction

Complex regional pain syndrome (CRPS) is a chronic pain condition characterized by “a continuing (spontaneous and/or evoked) regional pain that is seemingly disproportionate in time or degree to the typical course of pain after similar trauma or other lesion” [1]. The pain is regional and usually has a distal predominance of abnormal sensory, motor, sudomotor, vasomotor and/or trophic findings [1,2,3]. CRPS is usually triggered after fractures, sprains or surgery and most often affects limbs [4,5]. Due to this, people in the service industries suffer almost twice as often from CRPS as those in other occupations, possibly due to the physical activity associated with the job [6]. An estimated incidence of 5.46 and 26.2 per 100,000 person years according to studies conducted in 2003 and 2006 [7,8] has been reported. Recently, an incidence of 15 to 20 per 100,000 person years in Caucasian individuals has been assumed according to a population-based study [9]. In this study, 1043 patients with CRPS were analyzed during a study period of 20 years, thus showing the difficulty to recruit large numbers of patients [9]. Furthermore, CRPS is extremely debilitating and has significant repercussions on the quality of life of patients [10,11]. The difficulty in understanding its pathophysiology makes both its diagnosis and treatment a demanding task [12]. The disturbance of the sympathetic nervous system seems to be involved in CRPS [13] and some symptoms that patients present in the affected limb, such as edema and skin texture or sweating changes, can be compatible with an automatic nervous system dysfunction [2,14,15]. For this reason, achieving the suppression of pain in the early phases of the condition can be very meaningful for many patients [16,17].

Sympathetic nerve blocks are a widely performed procedure in the treatment of CRPS, as they may reduce some of the sympathetically mediated symptoms and ease the pain [18,19]. Their purpose is to disrupt the patient’s pain perception by interrupting the pain signal that sympathetic nerves send to the brain [20]. The injection of a local anesthetic drug (such as bupivacaine, lidocaine or ropivacaine) around the sympathetic ganglia temporarily alters their functions and provides pain relief [21,22]. When lower extremities are affected, lumbar sympathetic blocks (LSBs) are performed, blocking the lumbar sympathetic ganglia between lumbar vertebral levels L2 and L4 [23,24]. Lumbar sympathetic blocks have been questioned by several authors for the treatment of upper and lower limb CRPS [2,25,26]. According to a survey among 248 pain physicians, nearly 65% of the respondents consider sympathetic blocks moderately effective for treating CRPS [26]. However, some reports state that there is relatively weak evidence supporting their effectiveness in CRPS [2,27] LSBs have broadly been employed to treat many pain afflictions in the lower limbs and encouraging results have been described for CRPS [11,26,28], hyperhidrosis [29,30], herpes zoster [31], phantom limb [24] or diabetic neuropathy [32]. According to previous studies, LSBs are an efficient way to deal with sympathetic pain, especially in patients with lower extremity CRPS [33,34]. A double-blind, placebo-controlled study conducted in pediatric patients with CRPS revealed that LSBs produced a significant decrease in pain intensity compared to pretreatment values of allodynia and verbal pain score in the treatment group [34].

The lumbar sympathetic ganglia are located on the anterolateral aspect of the lumbar vertebral bodies [35,36]. Although their exact location, number and size are variable, four ganglia in each trunk are usually found [23,35]. The abdominal aorta lies anterior to the chain on the left and the inferior vena cava is located anterior to the chain on the right [23,36]. Some time ago, anatomic landmarks were used for guidance in most blocks but small inaccuracies in the needle placement could lead to complications because of the closeness of vital elements. For this reason, blocks are currently performed under image guidance given that a great precision of the needle placement is required. There is no standard to perform the guidance, although fluoroscopic guidance (FL) is most frequently used [26] as it provides great accuracy with a success rates of 67% [37,38]. However, other imaging techniques such as computerized tomography (CT) [39], magnetic resonance imaging (MR) [40] or ultrasound (US) [25,41] have also been described. When the fluoroscopy-guided approached is used, LSBs are often considered correctly performed when there is radioscopically confirmed contrast dye spread, i.e., forming a line conforming to the anterolateral margin of the vertebral bodies [25,42]. However, the variability of contrast spread may be subject to anatomic differences and/or secondary redistribution following injection [43] and an LSB under radioscopic guidance does not always ensure an exact performance. In order to evaluate the effect of an LSB, different methods are usually performed, including skin conductance response [39,44], laser Doppler flowmetry [45], skin plethysmography [46,47], perfusion index [48], skin temperature [49,50] and any combination of these methods.

In glabrous (non-hairy) skin, i.e., the sole of the feet and toes, arteriovenous anastomoses are found [51]. When LSBs are performed, the nerve supply is interrupted and changes in skin blood flow at the distal parts of the extremities occur [52,53]. For this reason, monitoring changes in skin temperature is used as an indicator of sympathetic block success [50,54]. Thus, LSBs have been considered successful when changes in the ipsilateral temperature between pre-block and post-block are ≥2 °C [24,37]. In the past, the common clinical practice of temperature assessment involved manual palpation of the foot. However, the temperature changes are typically too small to assess the temperature in various parts of the foot [55,56,57]. Several studies [41,44] have focused on monitoring changes in the skin temperature of the limbs to evaluate sympathetic blocks. To do so, temperatures were measured using skin probes (DM 852 Medical Precision Thermometer, Ellab A/S, Hilleroad, Denmark) attached to the middle of the plantar surface of the ipsilateral foot [46] or attaching adhesive thermocouple probes (accuracy of ±0.1 °C) (Solar 8000M, General Electric Healthcare, Milwaukee, WI, USA) to the plantar surface of the feet [37]. The problem of measuring skin temperature using probes is that this not only implies a much more laborious analysis, but also the attachment of thermocouples to the skin, which may alter thermal data [58]. Moreover, assessing only the temperature at a few points can be insufficient to determine whether the block is successfully performed or not [48,50].

Infrared thermography (IRT) is a non-invasive technique that, for biomedical purposes, is used to detect alterations in skin temperature by capturing the emitted radiation from a body [59]. Since it is performed at a distance from the body under study, it is harmless, and the skin temperature is not altered [60]. Moreover, it allows rapid recording of radiation data, which implies that a large amount of thermal information in real time is provided. Its features make it highly suitable to monitor temperature. For this reason, IRT has been applied in medicine for monitoring diseases and it has been broadly applied in several areas of biomedicine such as diabetic foot diagnosis, breast mass diagnosis or CRPS [61,62]. Some previous works have also analyzed the usefulness of measuring skin temperature by means of IRT in the assessment of sciatic nerve blocks [63], epidural anesthesia [64] and combined femoral and sciatic nerve blocks [65]. Although IRT can be a powerful instrument for assessing LSB performance, there is a lack of literature about this topic [65,66] and more studies are necessary to explore its usefulness.

The main objective of this work was to analyze the skin temperature data of the plantar foot acquired during the performance of lumbar sympathetic blocks in patients with complex regional pain syndrome affecting lower limbs. These preliminary results will evaluate the potential application of infrared thermography as a support technique in the assessment of lumbar sympathetic blocks.

## 2. Materials and Methods

### 2.1. Experimental Study

Forty-four lumbar sympathetic blocks (LBS) for the treatment of lower limbs CRPS in 13 patients (7 men) with an age of 41 ± 7 years old (mean ± standard deviation) were performed. Data were obtained between November 2019 and March 2020 at Hospital Intermutual de Levante (Valencia, Spain), which is an insurance company’s hospital where patients are usually workers. All procedures were performed by a team consisting of 1 or 2 pain medicine physicians.

The study was approved by the Ethics Committee of the Universitat de València (Reference: 1250779) and the participants signed the informed consent.

### 2.2. Lumbar Sympathetic Block Procedure

**Figure 1:**
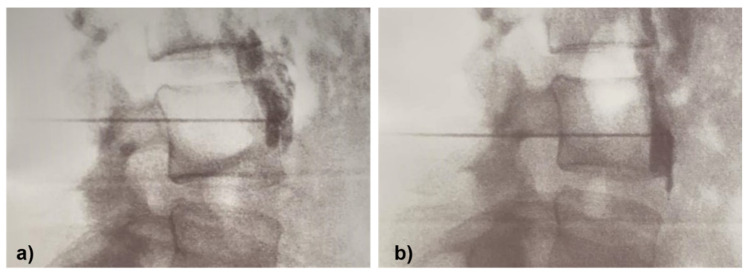
Lateral fluoroscopic view of the level of spread of the dye. (**a**) An LSB assessed with IRT when failed and (**b**) LSB after the repositioning maneuver of the needle.

For each patient, a series consisting of 3 “consecutive” (roughly 2 weeks apart) lumbar sympathetic blocks was scheduled, provided that each procedure was successful in the first instance (without needle relocation maneuvers). Otherwise, participants would go through more blocks as detailed in the next paragraph. In each procedure, patients were placed in the prone position with bare feet and their backs were sterilely prepared. The LSBs were guided by fluoroscopy and oblique, lateral and anteroposterior (AP) view images were obtained using a C-arm (Flexiview, General Electrical Medical System) to ensure proper site of entry (Figure 1). Although fluoroscopy is a common technique used to ensure the needle placement, images are 2D and for this reason the information provided is not always accurate enough [25,26]. Therefore, the blockade can be also monitored by other assessments such as the temperature increase in the extremity analyzed [37,50]. In this sense, IRT was used in this study to experimentally check whether a temperature increase occurred, as a complementary procedure. Therefore, before administering the injection of the local anesthetic (2 mL lidocaine 2%), a contrast dye was injected to confirm needle placement. Immediately after that, thermal images were captured of both feet soles. Participants were instructed to keep their feet still while thermal images were taken.

**Figure 2:**
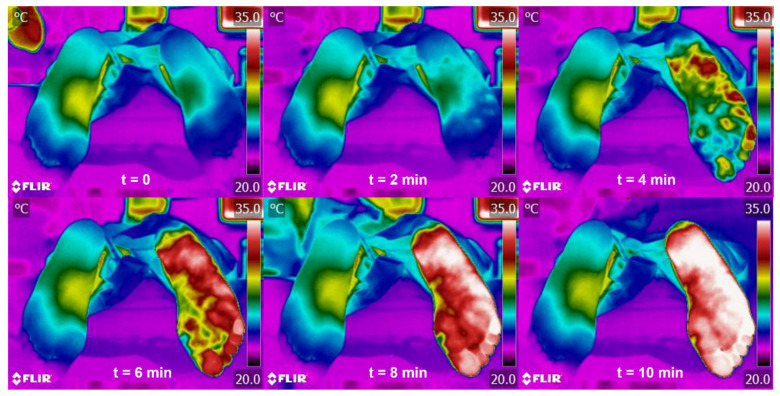
Infrared images of the soles of the feet on injecting of local anaesthetic (t = 0) and every 2 min thereafter (with the right foot being the ipsilateral).

When skin temperature changes in the ipsilateral sole were observed within 4 min (Figure 2), the procedure was considered responsive, and the medication was injected. On the other hand, when the procedure was determined as unresponsive, a repositioning maneuver of the needle was carried out and the process was repeated (from the lidocaine injection). In those cases, although the participant underwent more than 3 blocks in all, the medication was only injected for each successful block. Only when 3 consecutive repositioning maneuvers were performed, and though considering the procedure unresponsive, the medication was injected anyway to avoid complications in the participant.

### 2.3. Thermal Data Acquisition

Infrared thermography results can be influenced by several factors that can affect skin temperature. To avoid mistaken apparent temperatures, some procedures have been taken into account in concordance with literature recommendations [59,67]. Prior to entering the operation room, participants were asked to take off pants and shoes and they placed surgical booties on their feet. For a period of 15 min, they acclimatized to lying on a stretcher on which they were transferred to the operating room following the acclimatization period. When the participants arrived at the operating room, they were positioned in a prone position with bare feet and held that position for 15 min [68]. During this period, movements of the participant must be avoided as well as touching of their feet. After that, IRT acquisitions were performed in the same operating room with a controlled ambient temperature of 22.0 ± 0.5 °C and relative humidity 47 ± 5%.

**Figure 3:**
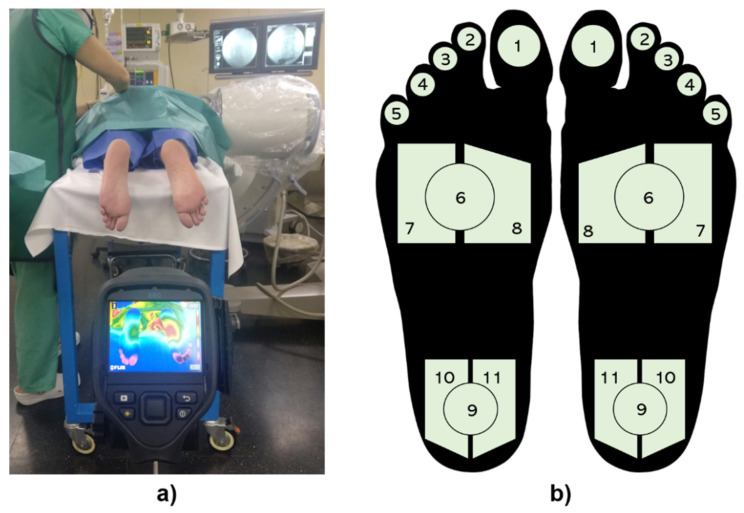
(**a**) Data acquisition setup during a lumbar sympathetic block performance (**b**) and the 11 ROIs selected in each foot: (1) Toe 1; (2) Toe 2; (3) Toe 3; (4) Toe 4; (5) Toe 5; (6) Central metatarsal; (7) Lateral metatarsal; (8) Medial metatarsal; (9) Central heel; (10) Lateral heel; (11) Medial heel.

Infrared data were acquired using the FLIR E60 thermal camera (FLIR Systems, Inc., Wilsonville, OR) with a pixel infrared resolution of 320 × 240, a field of view (FOV) of 25° × 19°, an instantaneous field of view (IFOV) of 1.36 mrad, a thermal resolution (NETD) of <50 mK at 30 °C and measurement uncertainty of ±2 °C of the overall temperature reading. The camera used in this study was checked before the experimental phase using a blackbody (BX-500 IR Infrared Calibrator, CEM, Shenzen, China) with target emissivity of 0.95, resolution of 0.1 °C and measurement uncertainty of ±0.8 °C. It is recommended to place the cameras as close as possible to the region to be measured to have the largest possible number of pixels, resulting in robust data [67]. For this reason, the minimum distance at which both plantar feet could be included in the same image was selected and the camera was mounted on a tripod at distance of 1.5 m from the participants’ feet and perpendicular to them, as shown in Figure 3a. Furthermore, the optical properties of the camera were considered. In this sense, a minimum object size of roughly 6 mm should be necessary to obtain an accurate temperature measurement at a distance of 1.5 m [69]. In this study, the minimum object to be analyzed corresponded to the little toe, which in the majority of cases exceeded this requirement. Emissivity was fixed at 0.98 for skin measurements [70]. The thermal camera was connected via USB to a laptop with the software FLIR Tools + (FLIR Systems, Inc., Wilsonville, OR, USA). Infrared images were acquired automatically every 10 s during the time required.

Measurements were made at the affected and contralateral extremities at baseline (right after the injection of the local anesthetic) and 10 min after the injection of the medication. During the LSB procedure, the pain physicians visualized the IRT video in real-time and classified the block depending on the observed thermal changes within the first 4 min in (a) failed (when no thermal changes were observed), (b) successful (when ipsilateral foot presented an increase in skin temperature) or (c) successful with contralateral increase (when both feet presented an increase in skin temperature). When no thermal changes were observed in the ipsilateral foot within the first 4 min after the lidocaine injection, a repositioning maneuver of the needle was then performed. Thus, in failed blocks, thermal images were recorded only for the first four minutes (starting from the lidocaine test). In successful blocks, thermal images were recorded from the baseline until 10 min after the medication was injected. In this sense, the duration of the thermal data acquisition depended on the ipsilateral skin temperature increase response speed.

### 2.4. Regions of Interest

Each plantar foot was divided into 11 regions of interest (ROIs) [55,71] shown in Figure 3b: ROIs 1 to 5 are the toes, ROIs 6 to 8 are the metatarsal areas of the foot and finally ROIs 9 to 11 are the ones situated on the heel of the foot. For each one of these regions, the mean temperature, the standard deviation (SD) and the maximum value were extracted for each frame using in-house software developed under MATLAB (The MathWorks, Inc., Natick, MA, USA). Mean skin temperature is one of the most used metrics, presenting advantages such as being both a representative value and able to remove the effect of punctual errors [72,73]. Maximum temperature is the highest value within an ROI, which can be associated with blood perfusion and inflammation [74]. Finally, SD of the ROI provides information about the data dispersion within the ROI, which could therefore be an indicator of an onset of blood perfusion [75].

Data extraction was performed by means of a semi-automatic algorithm. In this way, infrared images were first automatically segmented [76], and each plantar foot was divided into the 11 ROIs described above. Nevertheless, since feet movements occurred, some adjustments every few frames had to be done manually.

### 2.5. Statistical Analysis

Statistical analysis was performed using the software RStudio (version 1.2.5033, RStudio, Boston, MA, USA). A non-normal distribution of most of the thermal data was confirmed using the Shapiro–Wilk test (*p* < 0.05). Then, to assess the evolution of skin temperature over the measured period of time, a Kruskal–Wallis test with post hoc Wilcoxon test and Bonferroni correction was performed for each parameter (mean, maximum and SD skin temperature), each foot (ipsilateral and contralateral) and for each medical classification (failed, successful, successful with increase in the contralateral foot). Since the aim of our study was to detect an alteration in the thermal parameters over the recording time, the baseline time (0 s) was compared with the following times. In order to analyze which ROIs were more sensitive to this temperature increase, differences between two times (baseline and 240 s) were assessed in each ROI using a Kruskal–Wallis test with post hoc Wilcoxon test and Bonferroni correction for each parameter (mean, maximum and SD skin temperature) for the ipsilateral foot and for the successful group. Cohen effect size (ES) was calculated for the significant differences found in the pairwise comparisons and they were classified as small (0.2–0.5), moderate (0.5–0.8) or large (>0.8). Therefore, to detect a significant and considerable thermal alteration over the recording time, the level of significance was established at α = 0.05 and ES being moderate or large. Results are reported as mean with 95% confidence intervals (CI 95%), also presenting the CI 95% of the differences between conditions.

## 3. Results

### 3.1. Medical Classification in Real Time Using Infrared Thermography

Of the LSBs performed at the first attempt, 68.3% were considered by medical staff as successful on observing the skin blood flow vasodilation profile. LBSs were considered as failures when no thermal pattern alterations were observed after the first 4 min. A repositioning maneuver of the needle was then undertaken and the procedure repeated (lidocaine test along with the temperature evaluation). Following this, 53.8% of the repositioned LSBs performed were considered by medical staff to be successful. Among all the successful cases (in the first or posterior attempt in the same session), 19.5% were observed to also present temperature changes in the contralateral foot.

### 3.2. Quantification of Mean Skin Temperature

**Figure 4:**
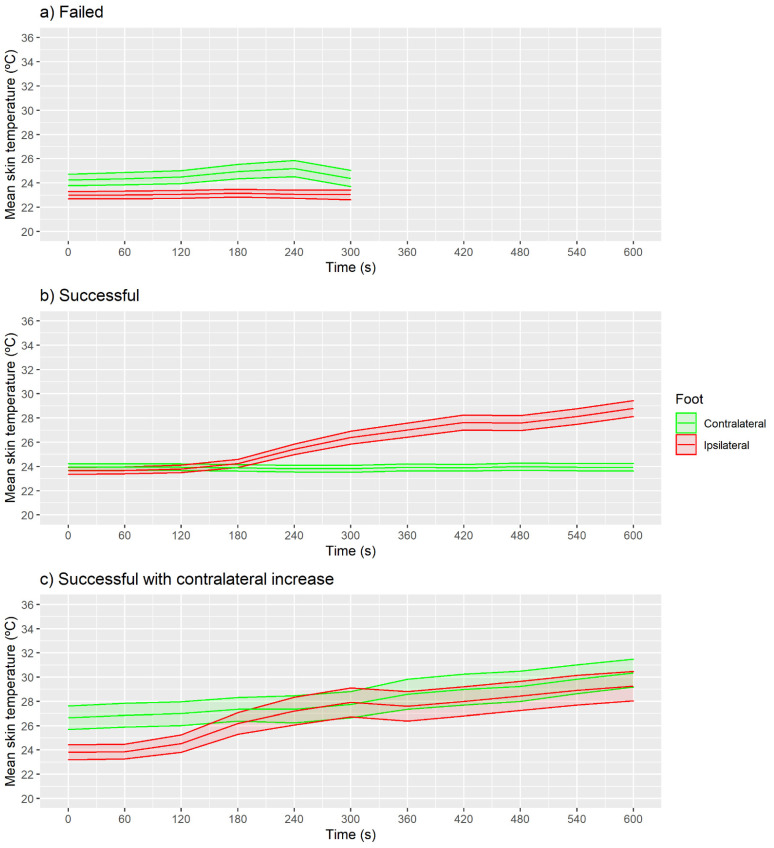
Evolution of mean skin temperature with shaded CI 95% area shown in the three classified groups using IRT in real time. (**a**) Failed, (**b**) Successful, (**c**) Successful with contralateral increase.

Figure 4 presents the mean skin temperature of the medical classification groups without considering the ROI factor. In the case of the failed group (Figure 4a), similar temperatures were observed in the two feet at all the times measured (*p* > 0.05 and ES < 0.5).

For the successful group (Figure 4b), 420 s was the first moment when the ipsilateral foot presented higher skin temperatures than at the baseline moment (0 s) (CI 95% of the difference (1.4, 2.1 °C), *p* < 0.001 and ES = 0.5). For this group, the contralateral foot presented similar temperatures between the baseline moment (0 s) and the following moments (*p* > 0.05 and ES < 0.5). In the case of the successful with contralateral increase group (Figure 4c), the first moment that the ipsilateral foot presented higher skin temperature than at the baseline moment was at 180 s (CI 95% (1.9, 3.3 °C), *p* < 0.001 and ES = 0.5), with even higher increases at 300 s (CI 95% (3.4, 5.2 °C), *p* < 0.001 and ES = 0.8). For this group, the contralateral foot presented higher skin temperatures at moment 540 s than at the baseline moment (CI 95% (1.6, 4.8 °C), *p* < 0.001 and ES = 0.5).

**Figure 5:**
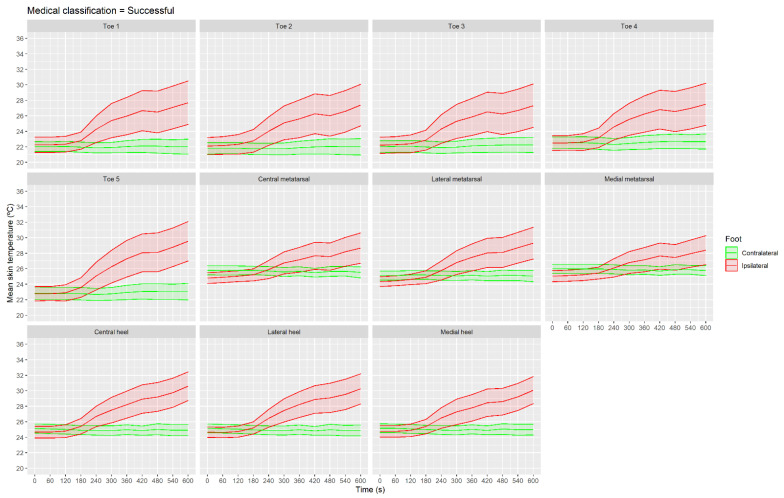
Evolution of mean skin temperature with shaded CI 95% area shown in the different regions of interest in the group classified as successful using IRT in real time.

Figure 5 presents the mean skin temperature data for all the ROIs of the successful group. Mean skin temperature at 240 s was higher in all the ROIs than at the baseline moment: toe 1 (CI 95% (0.5, 3.5 °C), *p* = 0.01 and ES = 0.7); toe 2 (CI 95% (0.6, 3.3 °C), *p* < 0.001 and ES = 0.6); toe 3 (CI 95% (0.6, 3.6 °C), *p* < 0.01 and ES = 0.7); toe 4 (CI 95% (0.7, 3.5 °C), *p* < 0.01 and ES = 0.7); toe 5 (CI 95% (0.7, 3.8 °C), *p* < 0.001 and ES = 0.8); central metatarsal (CI 95% (0.1, 2.1 °C), *p* = 0.01 and ES = 0.5); lateral metatarsal (CI 95% (0.3, 2.5 °C), *p* < 0.01 and ES = 0.7); medial metatarsal (CI 95% (0.0, 2.1 °C), *p* = 0.04 and ES = 0.5); central heel (CI 95% (0.9, 3.2 °C), *p* < 0.001 and ES = 0.9); lateral heel (CI 95% (0.8, 2.8 °C), *p* < 0.001 and ES = 0.9); and medial heel (CI 95% (0.6, 2.9 °C), *p* < 0.01 and ES = 0.8).

### 3.3. Quantification of Maximum Skin Temperature

**Figure 6:**
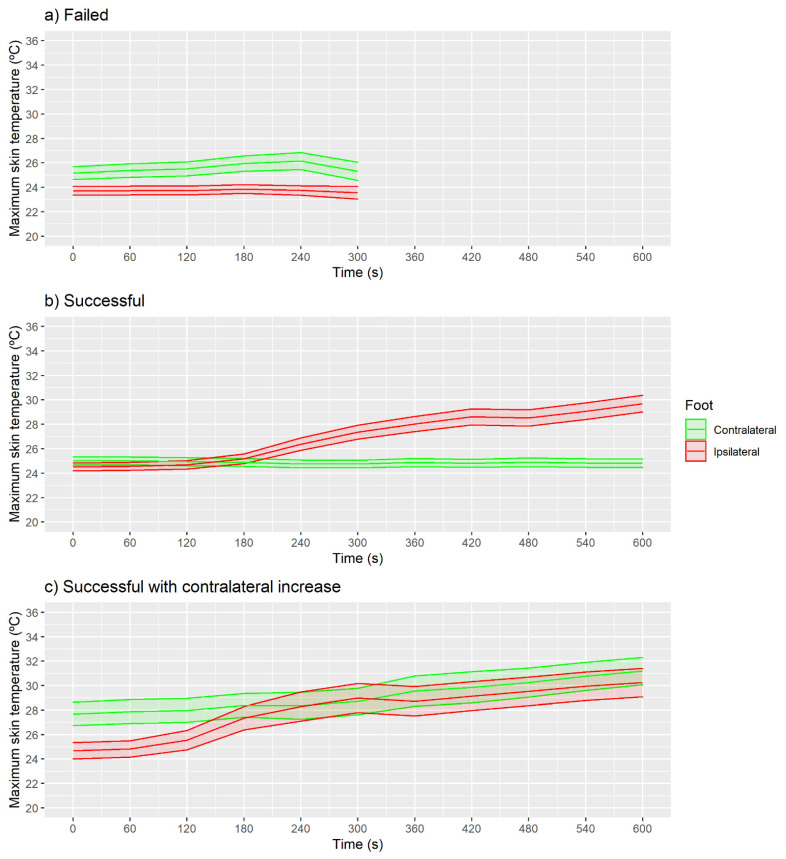
Evolution of maximum skin temperature with shaded CI 95% area shown in the three classified groups using IRT in real time. (**a**) Failed, (**b**) Successful, (**c**) Successful with contralateral increase.

For maximum skin temperature, no increase in skin temperature was observed in the failed group (Figure 6a; *p* > 0.05 and ES < 0.5).

For the successful group (Figure 6b), 300 s was the first moment when the ipsilateral foot presented higher maximum skin temperatures than at the baseline moment (CI 95% (2.3, 3.3 °C), *p* < 0.001 and ES = 0.6), with no increases on the contralateral foot (*p* > 0.05 and ES < 0.5). Similarly, as in the case of the mean temperature, in the successful with contralateral increase group (Figure 6c), the first moment was at 240 s (CI 95% (3.0, 4.6 °C), *p* < 0.001 and ES = 0.6). For this group, the contralateral foot presented higher maximum skin temperatures at moment 540 s than at the baseline moment (CI 95% (1.5, 4.7 °C), *p* < 0.001 and ES = 0.5).

**Figure 7:**
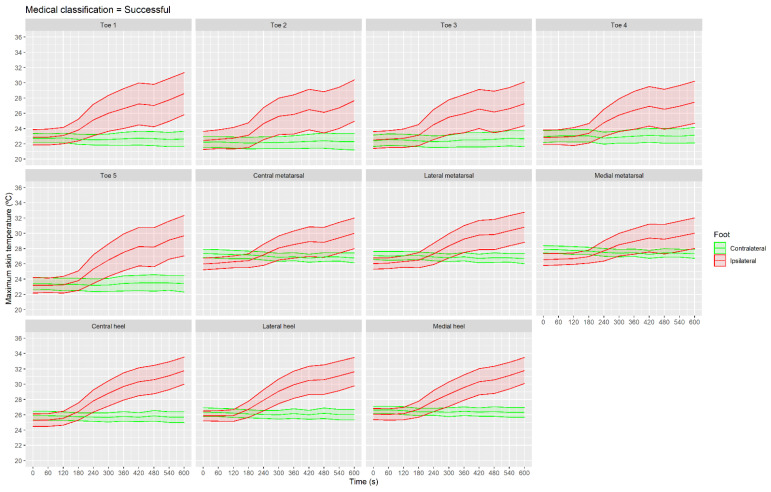
Evolution of maximum skin temperature with shaded CI 95% area shown in the different regions of interest in the group classified as successful using IRT in real time.

Figure 7 presents the maximum skin temperature for all ROIs of the successful group. Maximum skin temperature at 240 s was higher than at the baseline moment in toe and heel ROIs, but not in metatarsal ROIs (*p* > 0.05 and ES < 0.5): toe 1 (CI 95% (0.4, 4.1 °C), *p* = 0.03 and ES = 0.7); toe 2 (CI 95% (0.6, 3.8 °C), *p* < 0.001 and ES = 0.6); toe 3 (CI 95% (0.4, 3.6 °C), *p* < 0.01 and ES = 0.6); toe 4 (CI 95% (0.5, 3.4 °C), *p* = 0.01 and ES = 0.7); toe 5 (CI 95% (0.5, 3.4 °C), *p* < 0.01 and ES = 0.7); central heel (CI 95% (1.2, 3.8 °C), *p* < 0.001 and ES = 1.0); lateral heel (CI 95% (0.8, 3.3 °C), *p* < 0.01 and ES = 0.9); and medial heel (CI 95% (0.4, 3.0 °C), *p* = 0.01 and ES = 0.8).

### 3.4. Quantification of Standard Deviation Skin Temperature

**Figure 8:**
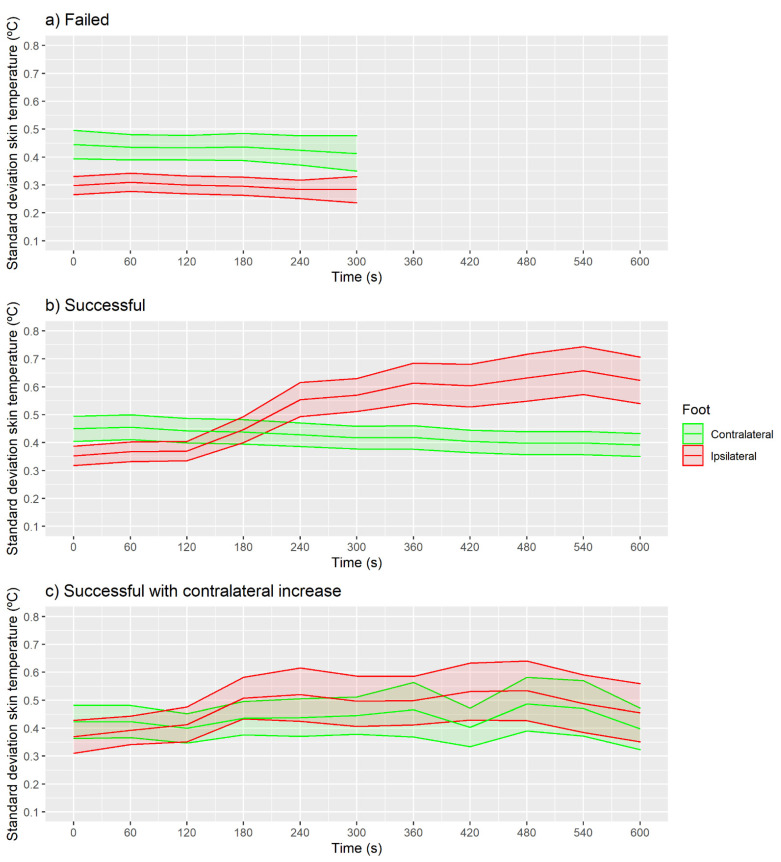
Evolution of standard deviation temperature with shaded CI 95% area shown in the three classified groups using IRT in real time. (**a**) Failed, (**b**) Successful, (**c**) Successful with contralateral increase.

The SD skin temperature was no different at baseline moment compared with the following moments in any of the feet for the failed and successful with contralateral increase groups (Figure 8a; *p* > 0.05 and ES < 0.5). For the successful group (Figure 8b), the 360 s moment presented higher SD skin temperature than baseline for the ipsilateral foot (CI 95% (0.2, 0.3 °C), *p* < 0.001 and ES = 0.5). The contralateral foot (Figure 8c) did not present any difference between baseline and the following moments (*p* > 0.05 and ES < 0.5).

**Figure 9:**
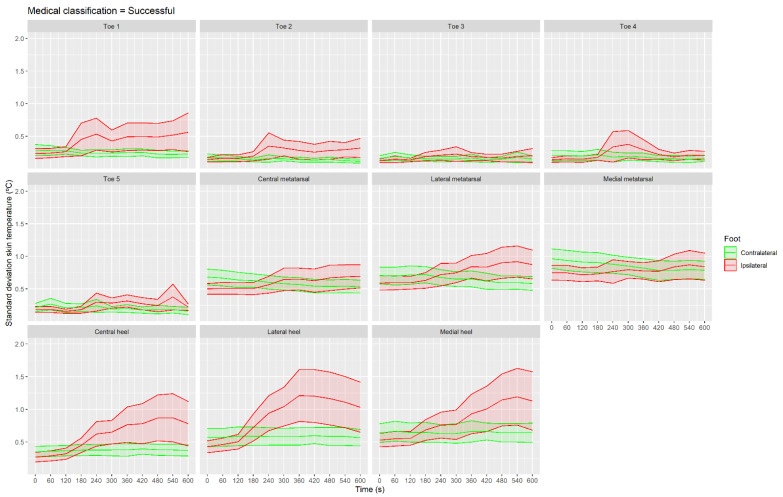
Evolution of standard deviation temperature with shaded CI 95% area shown in the different regions of interest in the group classified as successful using IRT in real time.

Figure 9 shows the SD skin temperature for all ROIs of the successful group. SD skin temperature at 240 s was higher than at the baseline moment in the first two toes and heel ROIs: toe 1 (CI 95% (0.1, 0.5 °C), *p* < 0.01 and ES = 0.9); toe 2 (CI 95% (0.0, 0.4 °C), *p* = 0.04 and ES = 0.8); central heel (CI 95% (0.2, 0.5 °C), *p* < 0.001 and ES = 1.2); lateral heel (CI 95% (0.3, 0.8 °C), *p* < 0.001 and ES = 1.3); and medial heel (CI 95% (0.0, 0.4 °C), *p* < 0.01 and ES = 0.7).

## 4. Discussion

Since this study was carried out in an insurance company’s hospital, the number of patients diagnosed with CRPS was greater than in other hospitals. However, we consider our study to be preliminary as a greater sample size is needed to provide sufficient statistical power to compare thermal data with clinical outcomes.

Lumbar sympathetic blocks have been questioned by several authors for the treatment of upper and lower limb CRPS [2,25,26]. The lack of clinic efficacy attributed in some cases to LSBs might be due to a lack of accuracy in the technique. Currently, there is no standard method to perform LSBs and different approaches among pain physicians can be found [38,77]. When fluoroscopy (FL) is used, LSBs are often considered correctly performed when there is radioscopically confirmed contrast dye spread. However, in some cases, this spread may be insufficient to confirm whether the needle tip reached the sympathetic chain [25,26]. In current clinical practice, there are different monitoring methods to assess the success of a sympathetic block. Among them, skin temperature measurement [50,78] can be performed by attaching thermocouple probes to the plantar surface of the feet [37,46]. However, this temperature measurement method presents some limitations such as possible data alteration due to inadequate contact with the skin [58,60] or insufficient thermal data to determine the block’s success properly [48,50]. In this sense, infrared thermography (IRT) provides much more information in real time, specifically, the IRT camera used in this study provides 76,800 temperature values (320 × 240 pixel infrared sensors). When performing an LSB, it may be advisable to evaluate skin temperatures on the plantar surface rather than on the dorsum of the foot since, there, a temperature increase has been shown to be less than two thirds that of the one obtained on the plantar surface [66]. Moreover, it has been suggested that skin temperature changes at the most distal parts of the extremity occur more reliably [50].

In view of the above and given that the set of thermal values using infrared thermography are depicted in an on-the-spot image, IRT enables physicians to check the thermal patterns taking place in real time on the soles of the feet and consequently to assess the block as failed or successful. In the present study, data confirmed the qualitative evaluation carried out by pain physicians, since the failed group did not present significant thermal changes in any of the variables analyzed. However, though thermal changes are assumed by medical staff to be detected within the first 4 min after lidocaine injection in the successful group, significant changes with at least a moderate effect cannot be detected until minute 7 for mean temperature, until minute 5 for maximum temperature and until minute 6 for SD. Taking this into account, if an automatic analysis were to be performed, waiting until minute 6 would be necessary. Regarding the successful with contralateral increase group, thermal changes have indeed been detected long before the successful group, specifically 3 and 4 min after the lidocaine test for mean and maximum temperature, respectively. The higher increase observed for this group and the lower SD (suggesting a more homogeneous increase throughout the foot) could indicate that this profile is related to a stronger reaction of the patient to lidocaine and, therefore, greater vasodilation. Future studies should try to explain this profile both from clinical and individual aspects.

Although most of the evaluated ROIs presented significant differences in any of the parameters, a large effect size between baseline and 4 min for mean, maximum and SD temperature was presented in the central and lateral heel. This behavior can be consistent with the blood flow distribution, since the blood supply to the plantar foot is primarily from the posterior tibial arteries [79]. However, the higher CI 95% observed when results differentiate the ROIs may suggest that there is an inter-participant variability in which ROI starts to heat up first. Therefore, we suggest that future studies should analyze all ROIs continuously.

Previous studies have evaluated skin temperature to determine the success of LSBs using temperature probes. One study reported that an LSB was considered successful if the rate of change reached more than 2 °C/min within 5 min after drug injection [37]. Another study showed that the onset time for obtaining a temperature increase of 2 °C or more in a successful LSB was 476.2 ± 112.6 s when the fluoroscopy guided approach was performed [38]. The CI 95% values of our results are in agreement with these previous studies. Moreover, the infrared data gathered in our study not only enable the location and positioning of the temperature changes within the foot in a precise manner, but also specify the moment at which these thermal changes occur. In this sense, thermographic intraprocedural control might be an instrument of great value in achieving better outcomes since it is a fast and precise technique to assess the success of performing blocks in the clinical setting.

For each patient, a series of three lumbar sympathetic blocks were performed two weeks apart. Nevertheless, the total number of blocks each participant underwent in the end depended on the successful or failed blocks. In this sense, only participants having three procedures classified as successful in the first instance, i.e., without a needle reposition maneuver, underwent three blocks. Otherwise, participants with some procedures classified as failed went through more blocks (with a limit of three consecutive failed blocks). Consequently, each participant had the medication injected only for each successful block, although previously he or she could have undergone some failed ones. Considering this, the number of failed blocks in a patient (nine maximum) could be related to the pain relief response. Thus, the influence of the LSB classification and patients’ clinical parameters such as pain relief should be deeply analyzed in future works in order to assess when LSBs are effective [22].

Finally, the human body is symmetric in the baseline condition of a healthy population, so it is considered that differences greater than 0.5 °C between sides could imply physiological dysfunctions [80,81,82]. In this sense, our study shows how in a basal condition, CRPS patients present a lower temperature in the ipsilateral foot. This lower temperature may be related to a lower skin blood flow produced by an automatic nervous system dysfunction in the affected limb of these patients [2,14].

There are some limitations to the present study. All LSBs were assessed with IRT, thus the absence of a control group could have improved the success rate. There are also some technical variations in performing the procedures, including the individual pain physician performing the LSB, or the volume of injectate. Inherent features in patients, such as the diverse morphology of feet and fingers along with shifts in the position of the feet make the analysis more inaccurate and time-consuming. To determine the degree and or duration of pain relief after LSBs in the treatment of CRPS, an in-depth analysis of the thermal patterns should be performed. Moreover, these profiles observed during the LSBs should also be correlated with the clinical outcomes. Finally, future studies should explore other image analysis methods and metrics such as tissue activity ratio [83,84] or the Tmax method [74].

## 5. Conclusions

The main objective of this preliminary study was to analyze the skin temperature data of the plantar foot measured during the performance of LSBs in patients with CRPS affecting the lower limbs. The use of infrared thermography in real time allowed the medical staff to determine whether the LSBs performed were successful, stating that 32% of the cases did not register temperature variations after lidocaine was injected, which forced them to relocate the position of the needle. For successful cases, after the injection of lidocaine, increases in foot skin temperature of a moderate effect size were observed at 420 s for the mean, at 300 s for the maximum temperature and at 360 s for the SD.

This paper evaluates thermal data of plantar feet in patients with complex regional pain syndrome who underwent lumbar sympathetic blocks. The preliminary results of our study show that measuring skin temperature in real time using infrared thermography is valuable in assessing the success of lumbar sympathetic blocks. As a non-invasive technique, with considerably feasible clinical applicability, it has great potential for improving procedural accuracy in the performance of lumbar sympathetic blocks and, therefore, in the achievement of better outcomes.

## Data Availability

The datasets generated and analyzed during the current study are available from the corresponding authors on reasonable request.
